# Association of TLR7 Variants with AIDS-Like Disease and AIDS Vaccine Efficacy in Rhesus Macaques

**DOI:** 10.1371/journal.pone.0025474

**Published:** 2011-10-13

**Authors:** Roman A. Siddiqui, Michael Krawczak, Matthias Platzer, Ulrike Sauermann

**Affiliations:** 1 German Primate Center, Leibniz Institute for Primate Research, Göttingen, Germany; 2 Genome Analysis, Leibniz Institute for Age Research–Fritz Lipmann Institute, Jena, Germany; 3 Institute of Medical Informatics and Statistics, Christian–Albrechts University, Kiel, Germany; University of Cambridge, United Kingdom

## Abstract

In HIV infection, TLR7-triggered IFN-α production exerts a direct antiviral effect through the inhibition of viral replication, but may also be involved in immune pathogenesis leading to AIDS. TLR7 could also be an important mediator of vaccine efficacy. In this study, we analyzed polymorphisms in the X-linked *TLR7* gene in the rhesus macaque model of AIDS. Upon resequencing of the *TLR7* gene in 36 rhesus macaques of Indian origin, 12 polymorphic sites were detected. Next, we identified three tightly linked single nucleotide polymorphisms (SNP) as being associated with survival time. Genotyping of 119 untreated, simian immunodeficiency virus (SIV)-infected male rhesus macaques, including an ‘*MHC* adjusted’ subset, revealed that the three *TLR7* SNPs are also significantly associated with set-point viral load. Surprisingly, this effect was not observed in 72 immunized SIV-infected male monkeys. We hypothesize (i) that SNP c.13G>A in the leader peptide is causative for the observed genotype-phenotype association and that (ii) the underlying mechanism is related to RNA secondary structure formation. Therefore, we investigated a fourth SNP (c.-17C>T), located 17 bp upstream of the ATG translation initiation codon, that is also potentially capable of influencing RNA structure. In *c.13A* carriers, neither set-point viral load nor survival time were related to the c.-17C>T genotype. In *c.13G* carriers, by contrast, the *c.-17C* allele was significantly associated with prolonged survival. Again, no such association was detected among immunized SIV-infected macaques. Our results highlight the dual role of *TLR7* in immunodeficiency virus infection and vaccination and imply that it may be important to control human AIDS vaccine trials, not only for MHC genotype, but also for *TLR7* genotype.

## Introduction

Toll-like receptor 7 (TLR7) localizes to intracellular vesicles in antigen-presenting cells such as plasmacytoid dendritic cells, macrophages, memory B cells and T cells. In these cells, TLR7 functions as a receptor for pathogen recognition in that it binds ligands like uridine-rich single-stranded (ss) RNAs or certain small interfering RNAs [1], [2], [3], [4]. Through the recognition of ssRNA derived from RNA viruses, TLR7 can also act as a danger receptor for viral infection (reviewed in [5], [6], [7]). Moreover, TLR7 activation modulates the innate and adaptive antiviral immune response by triggering the production of type I interferons, cytokines and chemokines [8], [9]. The X-linked *TLR7* gene appears to have been subject to positive selection during primate evolution, thereby underpinning its relevance in host-pathogen interaction [10].

TLR7 activation is an important mediator of vaccine efficacy. Targeting of TLR7 by synthetic agonists like polyUs21, or conjugation of TLR7 to a protein vaccine, can prime high-frequency polyfunctional type 1 T helper cell (Th1) and cytotoxic T-lymphocyte (CTL) response, probably through the activation of plasmacytoid dendritic cells (PDC) [11], [12], [13]. The stimulation of Toll-like receptors, in particular TLR7, also appears to be a determinant of the greater magnitude and Th1 polarization of the immune response induced by inactivated whole-virus H5N1 influenza vaccine, as compared to split or subunit vaccine-induced responses [14]. Furthermore, IGA responses important for mucosal immunity potentially also depend upon TLR7 activation [15]. Targeting TLR7 may therefore improve vaccine efficacy.

In HIV infection, TLR7-triggered IFN-α production appears to be a double-edged sword [16]. On the one hand, stimulation of antigen-presenting cells by synthetic TLR7 agonists like imidazoquinoline compounds augment HIV-1-specific T cell responses *in vitro* [17]. Moreover, administration of IFN-α or triggering of TLR7/8 by imidazoquinoline compound R848 has direct anti-HIV effects by inhibiting viral replication *in vitro*, and potentially *in vivo*, through multiple pathways [18], [19], [20], [21], [22], [23]. On the other hand, TLR7-triggered pathways appear to be directly involved in immune pathogenesis leading to AIDS [24], [25]. Chronic immune stimulation via TLR7 in mice caused progressive lymphoid system destruction resembling HIV-mediated immunopathology [26]. Opposing effects of TLR7 have also been observed in the context of respiratory syncytial virus infection (RSV) in mice. Whereas administering TLR7/8 agonists during immunization did hardly influence the phenotype upon challenge with RSV, the immunostimulatory properties of the same agonists increased disease severity when used in mice that had already been infected [27]. Finally, since IFN-α production is particularly enhanced in females upon TLR7 stimulation, the latter may also contribute to sex-specific disease progression [24], [28].

The pathophysiological role of TLR7 can be studied in some detail in humans owing to a functional polymorphism located in the signal peptide. This Q11L amino acid replacement (*rs179008*) has been shown to confer susceptibility to asthma [29] and to influence the course of hepatitis C infection [30], HIV disease progression and potentially HIV-1 acquisition [31]. Peripheral blood mononuclear cells (PBMCs) from individuals carrying the minor allele (11L) secreted less IFN-α upon stimulation by TLR7 agonist Imiquimod compared to PBMCs from individuals carrying the major allele [31]. Interestingly, the allele associated with diminished *ex vivo* IFN-α secretion was also associated with an accelerated time to onset of antiviral therapy (CD4 counts>350 µl) and higher set-point viral load in HIV-infected patients [31], indicating that the protective antiviral effects of TLR7 may prevail in the initial and chronic phases of infection.

The important role of TLR7 in viral infection and immunization prompted us to characterize *TLR7* gene polymorphisms in simian immunodeficiency virus (SIV)-infected rhesus macaques (*Macaca mulatta*), which represent the most important animal model of HIV infection. Moreover, the expression patterns of TLR7 and other TLRs in antigen-presenting cells such as dendritic cells, monocytes and B cells are similar in macaques and humans, but differ markedly from the expression profiles in mice [32]. TLRs also respond to the same ligands in macaques as in humans [32]. Upon targeted *TLR7* re-sequencing in 36 rhesus macaques of Indian origin, we detected 12 polymorphisms and subsequently investigated their possible association with AIDS-free survival and viremia in SIV-infected animals. We identified two polymorphisms, located immediately upstream and downstream of the *TLR7* translation initiation codon, to be associated with survival time and/or set-point viral load in untreated SIV-infected macaques. Interestingly, no such association was evident in immunized-infected macaques.

## Results

### Variability of the rhesus *TLR7* gene

Like in humans and many other species, the X-linked *TLR7* gene in rhesus macaque contains three exons, namely two untranslated exons encompassing the 5′ untranslated region (5′UTR) and the ATG translation initiation codon, and exon 3 spanning the sequence coding for the leader peptide, the mature TLR7 polypeptide and a 3′ untranslated region (3′UTR) of about 1700 bp not yet annotated firmly in rhesus macaque. In contrast to the human situation, however, no polymorphisms in the rhesus *TLR7* gene were known prior to this study. We therefore had to establish a panel of polymorphisms first before we could assess the impact of *TLR7* gene variability upon the progression and outcome of SIV infection in this species.

Initial DNA sequence analysis of all three exons and some intronic parts of the *TLR7* coding region, comprising a total of 7853 bp, was performed in a pilot sample 36 animals. Twelve variable positions were identified in the DNA sequence encoding the *TLR7* mRNA (Figure S1), four of which were located in the protein-coding region of *TLR7* (Figure S2). Two of the coding polymorphisms were non-synonymous whilst two were synonymous. The rhesus TLR7 amino acid sequence differs from human TLR7 by 19 amino acid substitutions (Figure S2). Notably, the amino acid sequences encoding the transmembrane and the Toll/IL-1 receptor (TIR) domain important for transport and associated signalling are identical in humans and rhesus macaques, and no polymorphisms were detected in these regions (Figure S2). Seven of the eight non-coding polymorphisms were located in the 3′UTR of exon 3 whereas one was found in exon 2, 17 bp upstream of the ATG translation initiation codon. Since the rhesus genome is not yet annotated as thoroughly as the human genome, we provisionally labelled the SNPs following the recommendations for the description of DNA sequence variants made by the Human Genome Variation Society [33]. Allele frequencies observed among the 36 macaques of the pilot sample are given in Table 1.

**Table 1:** Major allele frequencies (MAF) of 12 *TLR7* gene polymorphisms in 36 rhesus macaques of Indian origin.

Polymorphism	Genomic Position	MAF
c.-17 C>T	1056561	0.74
c.13 G>A (V5M)	10583408	0.68
c.204 A>G (T68A)	10583597	0.56
c.1710 T>A (V570V)	10585105	0.69
c.2352 G>A (R784R)	10585747	0.52
c.*437 G>A	10586982	0.94
c.*236 A>G	10587281	0.50
c.*867 T>C	10587412	0.82
c.*908_1911delGGCT	10587453	0.94
c.*941 A>G	10587483	0.81
c.*1604 C>T	10588146	0.69
c.*1681 A>G	10588222	0.85

Four single nucleotide polymorphisms (SNPs) were also genotyped in a larger sample. Thus, a total of 237 animals of Indian origin (46 females, 191 males), including the 36 samples used in the re-sequencing experiment, were investigated for SNPs c.-17C>T (5′UTR), c.13G>A (V5M), c.1710T>A (V570V) and c.*1604C>T (3′UTR). The last three polymorphisms were in strong pair-wise linkage disequilibrium (LD) in our sample (all pair-wise allelic r^2^>0.90) so that the respective allele frequencies were very similar (Table 1). Allele frequencies also differed hardly between the initial re-sequencing panel and the larger validation sample (data not shown).

### Association of *TLR7* polymorphisms c.13G>A, c.1710T>A and c.*1604C>T with survival time and viral load in untreated SIV-infected rhesus macaques

The initial re-sequencing experiment was carried out on 36 unrelated SIV-infected rhesus macaques that displayed the whole spectrum of disease progression (i.e., AIDS-free survival from 6 to >580 weeks post infection). Three of the discovered SNPs, namely c.13G>A, c.1710T>A, and c.*1604C>T, were suggestive of an association with survival time in the pilot sample. For further validation, a larger sample of 237 SIV-infected macaques was genotyped for the three SNPs either by Sanger sequencing or using a TaqMan-based allelic discrimination assay. In the following, we will only report upon c.13G>A, because the other two SNPs were in strong LD with this polymorphism (see above).

**Figure 1:**
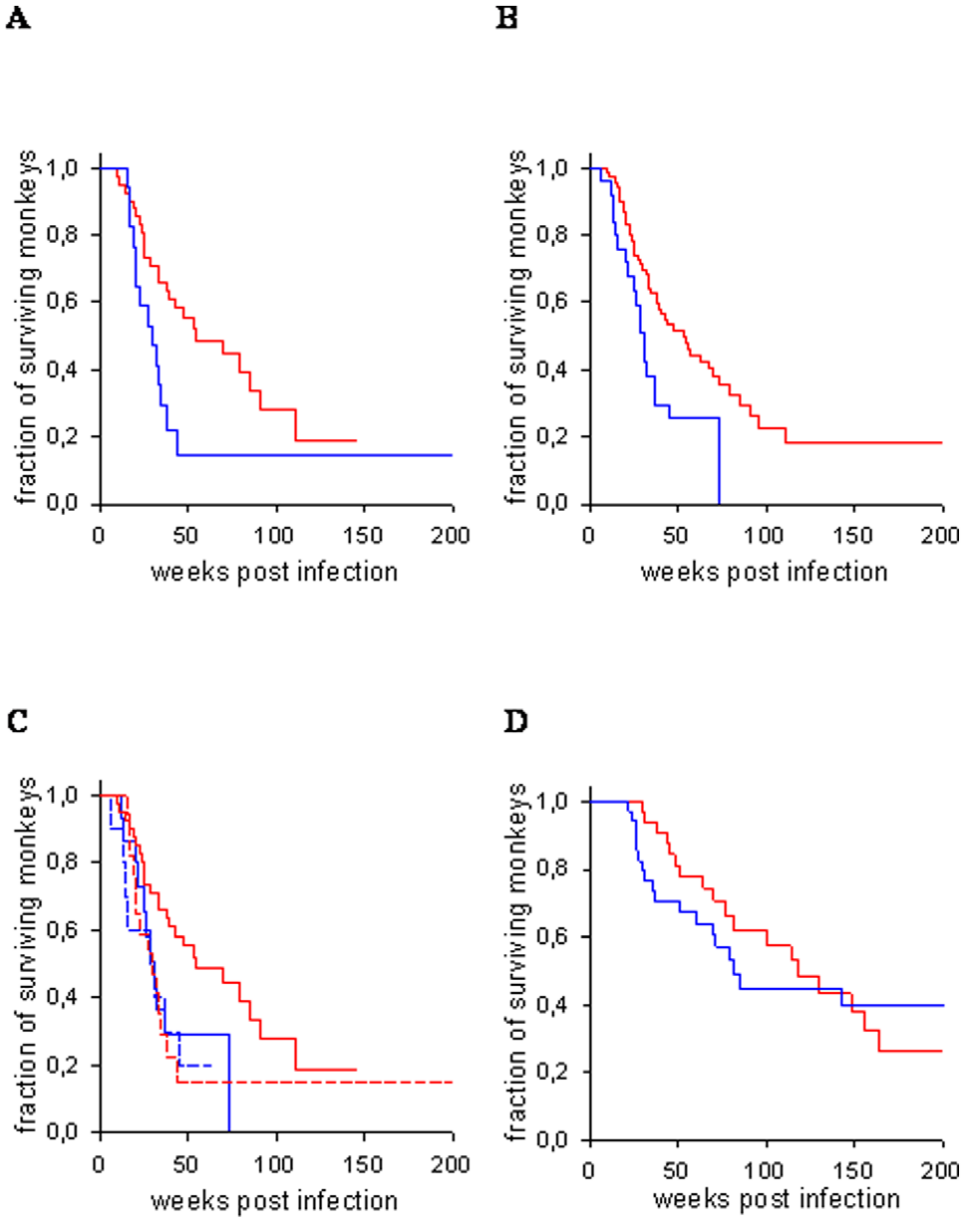
Kaplan-Meier curves of AIDS free survival of SIV-infected male rhesus macaques carrying allele *c.13A* (blue line) or *c.13G* (red line) of *TLR7* SNP c.13G>A. A: untreated-infected macaques (*c.13A*: n = 35, *c.13G*: n = 84) (p = 0.025); B: untreated-infected macaques after *MHC* adjustment (*c.13A*: n = 26, c.13G: n = 69) (p = 0.077); C: untreated-infected macaques after *MHC* adjustment carrying haplotype *c.-17T,c.13A* (blue discontinuous line, n = 14), *c.-17C,c.13A* (straight blue line, n = 11), *c.-17T,c.13G* (red discontinuous line, n = 18), *c.-17C,c.13G* (straight red line, n = 51); (c.13G carriers: *c.-17C>T*: p = 0.017); D: immunized-infected macaques carrying *c.-17C,c.13A* , *c.-17T,c.13A* or *c.-17T,c.13G* (blue line, n = 38) or *c.-17C,c.13G* (red line, n = 34) (p>0.3).

**Figure 2:**
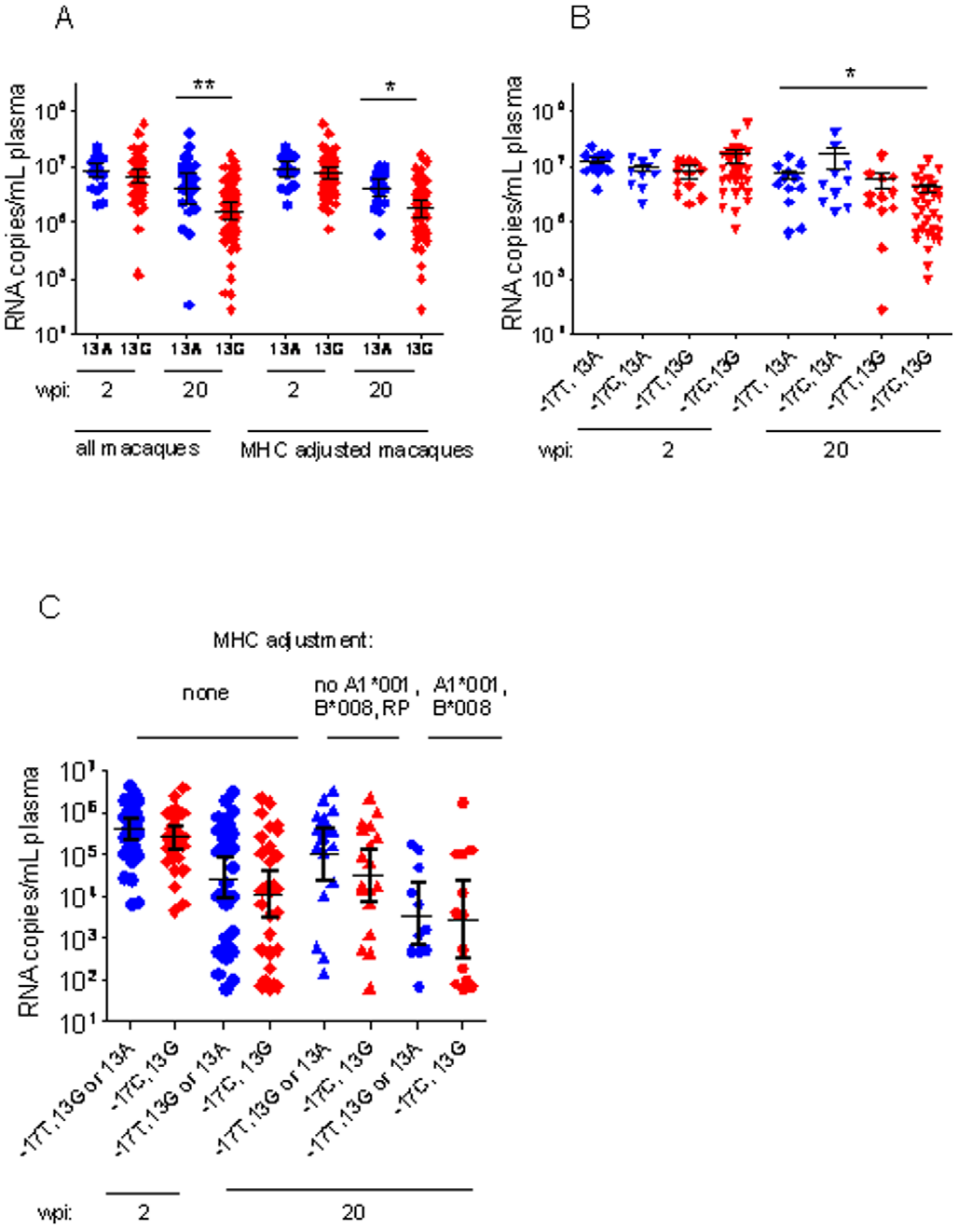
Plasma RNA copy number at peak (2 wpi) and set-point (20 wpi) of SIV-infected male rhesus macaques, stratified by the *TLR7* genotype. Geometric mean and 95% confidence interval are indicated by error bars. Significant differences are marked by asterisks. A: untreated SIV-infected carriers of allele *c.13A* (n = 23) or *c.13G* (n = 54), and untreated SIV-infected monkeys after *MHC* adjustment carrying *c.13A* (n = 17) or *c.13G* (n = 46). B: untreated SIV-infected rhesus macaques stratified by their joint c.-17C>T and c.13G>A genotype: *c.-17C,c.13A* (n = 12), c.-17T,*c.13A* (n = 10), *c.-17T,c.13G* (n = 12), *c.-17C,c.13G* (n = 34). C: plasma RNA copy numbers of immunized SIV-infected macaques carrying *c.13A* or *c.-17T,c.13G* (n = 33), *c.-17C,c.13G* (n = 31), *MHC* adjusted: *c.13A* or *c.-17T,c.13G* (n = 20), *c.-17C,c.13G* (n = 18), carrying *MHC* alleles associated with slow disease progression: *c.13A* or *c.-17T,c.13G* (n = 11) , *c.-17C,c.13G* (n = 10).

Among the 119 untreated-infected male macaques genotyped, AIDS-free survival was found to be significantly associated with the c.13G>A hemizygous status. Thus, male macaques carrying the minor allele (*c.13A*) progressed faster to AIDS than macaques carrying the major allele (*c.13G*; log-rank χ^2^ = 5.04, 1 d.f, p = 0.025) (Fig. 1A). Furthermore, c.13A carriers had a significantly higher viral load at set-point (F = 6.82, p = 0.011) (Fig. 2A). No difference in peak viremia at week 2 post infection was observed, indicating that initial viral replication is not strongly influenced by c.13G>A genotype (F = 1.33, p = 0.252).

Since the *MHC* genotype is known to influence both survival time and viral load in HIV-infected humans as well as in SIV-infected macaques, we controlled our analysis for the presence for *MHC* genotypes known to strongly influence disease progression (Figure S3). Inspection of the data revealed that *MHC* genotypes previously found to be associated with either rapid or slow disease progression were not evenly distributed among the male animals studied here [34]. In the group of c.13A carriers, *MHC*-genotypes associated with rapid disease progression were significantly overrepresented compared to c.13G carriers (17.1% vs. 2.4%; Fisher's exact test p = 0.008) whereas *MHC* genotypes associated with slow disease progression (Mamu-A1*001, -B*008) were underrepresented (8.3% vs. 20.2%), although the second difference failed to reach statistical significance (Fisher's exact test, p = 0.389). After excluding all of these animals from the analysis (‘MHC adjustment’), survival times were no longer significantly different between the two c.13G>A genotypes (log-rank χ^2^ = 4.14, 1 d.f., p = 0.077) but still showed a trend towards longer survival being associated with *c.13G* (Fig. 1B). However, even in the smaller, MHC-adjusted sample comprising 63 monkeys, *c.13A* carriers still had a significantly higher set-point viral load than *c.13G* carriers (F = 6.02, p = 0.017) whilst peak viral load did not differ between genotypes (F = 0.61, p = 0.439).

We also investigated 28 untreated SIV-infected females available to seek evidence for a potential sex difference in terms of the observed genotype-phenotype relationship. Whilst only one female was homozygous for *-c.13A*, *c.AG* heterozyotes and *c.GG* homozygotes were almost equally frequent (13 vs. 14). The relevant MHC genotypes were evenly distributed between both groups and no significant difference in AIDS-free survival time was apparent between *AG* and *GG* females (log-rank χ^2^ = 0.155, 1 d.f., p = 0.694). Further analyses were not deemed meaningful in view of the small sample size.

### *TLR7* polymorphism at position −17 (c.-17C>T) influences set-point viral load and survival time in *c.13G* carriers

Polymorphisms close to the ATG start codon can affect translation initiation and thus the protein expression. We therefore examined the potential effect of the c.-17C>T polymorphism, located in exon 2, which is not in linkage disequilibrium with the polymorphisms in exon 3. Exon 2 of the *TLR7* gene is remarkably conserved between human and rhesus macaque, and no polymorphisms have been detected in the respective human sequence so far. Since the polymorphisms at position −17 and +13 might both alter the local RNA secondary structure, we analyzed the phenotypic effect of the −17 polymorphism separately in both, *c.13A* and *c.13G* males. Among the 34 *c.13A* carriers, the c.-17C>T status was not significantly associated with either survival time, set-point viral load (both p>0.5) or peak viremia (p>0.1). From this analysis, only a single long-term non-progressing monkey carrying Mamu-A1*001 and -B*017 was excluded because all other *MHC* genotypes were almost identically distributed within the two c.13G>A genotype-defined groups (Figures 1C, 2B). Note, however, that only *MHC* adjusted monkeys are shown in Figures 1C and 2B in order to allow direct comparison with Figures 1B and 2A. *MHC* adjustment reduced the number of monkeys analysed but did not change the results of the statistical analysis.

In *c.13G* carriers, by contrast, the c.-17C>T polymorphism was significantly associated with survival time (log-rank χ^2^ = 5.66, 1 d.f., p = 0.017), but not with set-point or peak viral load (both p>0.5). Only *MHC* adjusted monkeys were included in this analysis because of the overrepresentation of slow progressor genotypes among *c.-17C,c.13G* carriers. Interestingly, survival time (p>0.6) and set-point viral load (p>0.1) did not differ significantly between *c.13A* and *c.-17T,c.13G* carriers, but between *c.13A* and *c.-17C,c.13G* carriers (survival time: log-rank χ^2^ = 6.21, 1 d.f., p = 0.013; set-point viral load: F = 8.10, p = 0.006). Thus, the inclusion of c.-17C>T genotype was capable of refining the genotype-phenotype association of c.13G>A. Eventually, only the *c.-17C,c.13G* haplotype turned to be significantly associated with prolonged survival and low set-point viral, compared to the other haplotypes (see Figures 1C, 2D).

### No association of *TLR7* polymorphisms with survival time and viral load in immunized SIV-infected macaques

Next, we analyzed the influence of the *TLR7* SNPs upon AIDS-free survival and viral load in 72 immunized-infected male monkeys. These animals were analyzed separately because their survival time was significantly prolonged and their set-point viral load significantly reduced compared to untreated-infected macaques (both p<0.001). This difference remained significant even after *MHC* adjustment (data not shown). The macaques were immunized with different vaccine formulations, but the majority of the respective vaccination trials included adenovirus-based vectors [35], [36], [37], [38]. In all experiments, peak viremia was lower among immunized than among untreated-infected macaques. Reduced peak viremia has been found to be associated with prolonged survival and reduced set-point viral load before, probably reflecting the preservation of central memory CD4^+^ T cells [35], [39].

Owing to the relatively small number of immunized male monkeys available in this study, we did not initially perform any *MHC* adjustment although rapid progressors, as predicted by their *MHC* genotype, were underrepresented among *c.13G* carriers (2.3%) compared to *c.13A* carriers (11%). In the immunized-infected macaques, neither AIDS-free survival (log-rank χ^2^ = 0.33, 1 d.f., p = 0.564), nor set-point viral load (F = 0.23, p = 0.636) nor peak viremia (F = 0.32, p = 0.574) were found to be significantly associated with c.13G>A genotype. Subsequent *MHC* adjustment led to insignificant results as well (all p>0.3). Immunized macaques carrying *TLR7* genotypes associated with more rapid disease progression in untreated -infected animals (i.e. *c.13A* and *c.-17T,c.13G*) did also not differ from carriers of *c.-17C,c.13G* (i.e., the haplotype associated with slow disease progression) neither in terms of survival time nor viral load,, and even after MHC adjustment (Figures 1D, 2C).

A trend towards prolonged survival was observed in immunized-infected female macaques compared to untreated-infected females (log-rank χ^2^ = 3.31, 1 d.f., p = 0.069). However, owing to the small sample size (n = 18), a detailed statistical analysis of the genotype-phenotype relationship was not considered warranted.

### Prediction of RNA secondary structure

**Figure 3:**
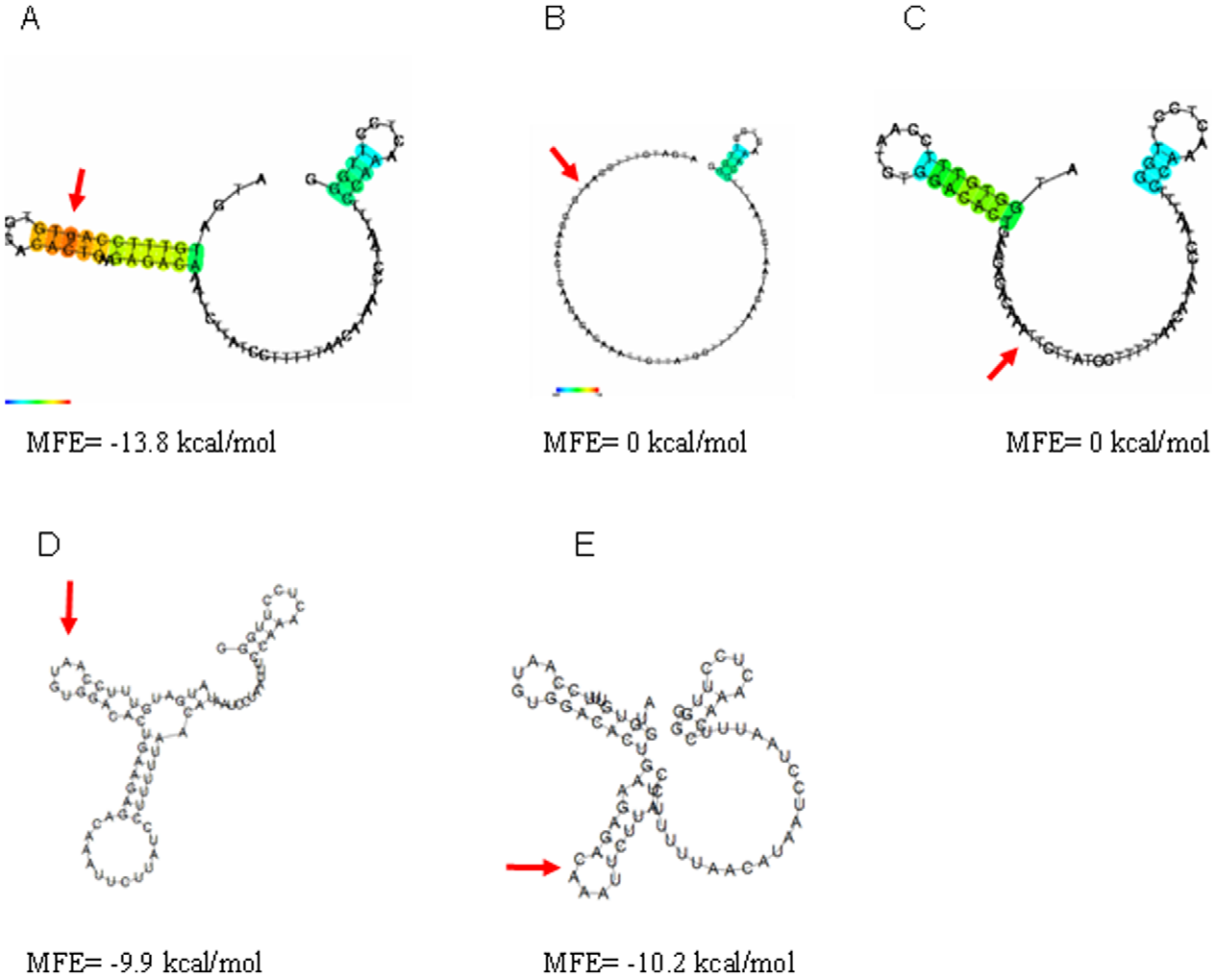
RNA secondary structure. RNA secondary structure as predicted by CentroidFold [36] of rhesus *c.13G* (A), rhesus *c.13A* (B), human (C) signal peptide-encoding sequences, corresponding to amino acid positions 1–26. Each predicted base pair is coloured according to a gradient (from blue to red) corresponding to the base-pairing probability (from 0 to 1). The heat colour can be interpreted as a confidence measure of the predicted base-pairing. Red arrows indicate the polymorphic positions in the respective signal peptide DNA sequence. Plain RNA secondary structures as predicted by RNAfold [37] are shown for rhesus *c.13A* (D) and human (E) signal peptide-encoding sequences. Both programs predicted identical RNA secondary structures for rhesus *c.13G*. The minimal free energy (MFE) of each structure is given.

Since RNA secondary structure is a major determinant of translation kinetics and efficiency, we analysed the potential effects upon RNA secondary structure of the SNPs associated with AIDS-free survival. Of the three tightly linked SNPs in exon 3, only c.13G>A, but not the two other SNPs, was found to influence RNA secondary structure (CentroidFold program) or to change the minimum free energy by more than −2.1 kcal/mol (RNAfold, Vienna Websuite). For the signal peptide-encoding sequence (position 1–78), both prediction programs calculated an identical RNA secondary structure for the major *c.13G* allele, with a minimum free energy (MFE) of −13.8 kcal/mol (Fig. 3A). The *c.13A* allele disrupts this structure. The CentroidFold program predicted no strong secondary structure (MFE = 0 kcal/mol) at all for this sequence (Fig. 3B) whereas the Vienna RNA software predicted a secondary structure with an MFE of −8.9 kcal/mol. Despite some minor differences, both programs therefore suggested a destabilisation of the secondary structure of TLR7 signal peptide RNA sequence by the *c.13A* allele. The c.-17C>T polymorphism in exon 2 is located in a region critical for ribosomal entry and also influences RNA secondary structure. Both programs predict a stronger secondary structure for *c.-17C* compared to *c.-17T* containing RNA (MFE Centroidfold: −12.7 vs. 0 kcal/mol, RNAfold: −20.2 vs. −17.6 kcal/mol). Analyzing the effect of the c.-17C>T polymorphism in *c.13A* and *c.13G* carriers individually revealed that the folding effects of the two polymorphisms may be interdependent. Centroidfold predicted an MFE of −39.7 kcal/mol for the RNA sequence spanning exons 1 and 2 and the signal peptide-encoding region in the case of the c.-17C,*c.13G* haplotype whereas the structures of the other haplotypes had an MFE between −30.0 and −29.1 kcal/mol (Table 2). RNAfold predicted an MFE of −51.5 kcal/mol for the *c.-17C,c.13G* haplotype and of −49.7 to −47.0 kcal/mol for the other three haplotypes. Restriction of the *in silico* analysis to smaller RNA strands (25 bases up- and downstream of the c.-17 and c.13 position) yielded similar results (Table 2). Here, the *c.-17C,c.13G* haplotype also had the highest MFE , and the other haplotypes had a lower but relatively similar MFE. Despite some quantitative discrepancies, both programs therefore suggested (i) a joint effect of positions −17 and 13 on the RNA secondary structure and (ii) an RNA secondary structure that is most stable for the *c.-17C,c.13G* haplotype.

**Table 2:** Minimal free energy (MFE) of RNA secondary structures.

SNP	Nucleotides analyzed^1^	MFE (kcal/mol) Centroidfold^2^	MFE (kcal/mol) RNAfold^3^
c.-17C	c.-98 to c.-1^4^	−12.74	−20.24
c.-17T	c.-98 to c.-1^4^	0	−17.64
c.13G	c.1 to c.78^5^	−13.8	−13.8
c.13A	c.1 to c.78^5^	0	−9.9
c.-17C,c.13A	c.-139 to c.78^6^	−29.96	−49.66
c.-17C,c.13G	c.-139 to c.78^6^	−39.66	−51.56
c.-17T,c.17A	c.-139 to c.78^6^	−29.13	−47.0
c.-17T,c.13G	c.-139 to c.78^6^	−29.70	−49.0
c.-17C,c.13A	c.-47 to c.38	−14.89	−14.89
c.-17C,c.13G	c.-47 to c.38	−17.69	−20.44
c.-17T,c.13A	c.-47 to c.38	−15.89	−15.89
c.-17T,c.13G	c.-47 to c.38	−14.6	−17.84

1 Nucleotide numbering refers to the cDNA sequence.

2 Reference 36.

3 Reference 37.

4 Corresponding to exon 2 sequence.

5 Corresponding to signal peptide encoding sequence.

6 Corresponding to exon 1, exon 2 and signal peptide encoding sequence.

For comparison, we also analyzed the potential RNA secondary structure of the human *TLR7* signal peptide-coding sequence. CentroidFold predicted an RNA secondary structure with an MFE of “0.0 kcal/mol” (Fig. 3C), whereas RNAfold predicted a structure with an MFE of −10.2 kcal/mol, indicating that the human *TLR7* signal peptide-coding RNA structure is probably more similar to that of the rhesus *c.13A* allele. Both programs predicted that the Q11L (rs179008) polymorphism, which has been reported to influence viral load and time to AIDS in humans, does not influence the secondary structure of the human TLR7 signal peptide-coding RNA [31].

Finally, we investigated the influence, upon the potential RNA structure, of those SNPs that were not associated with AIDS-free survival in the initial sample of 36 macaques. The two remaining coding polymorphisms and three of the six polymorphisms in the 3′UTR did not change the predicted RNA secondary structure. The polymorphisms at position c.*236, c.*867 and the insertion/deletion c.*908_1911delGGCT changed the calculated MFE by more than 3 kcal/mol. None of polymorphisms in the 3′UTR was located close to a likely functional element [40]. Notably the polymorphisms at position c.*867 and c.*908_1911delGGCT flank the site of another functional polymorphism (TLR7 3′UTR SNP, rs3853839) reported in the human *TLR7* gene [41].

## Discussion

Prior to this study, no polymorphisms in the *TLR7* gene locus of rhesus macaques were known. We therefore re-sequenced the *TLR7* gene in 36 rhesus macaques of Indian origin and identified 12 polymorphisms. An initial analysis of 36 monkeys revealed three tightly linked SNPs to be associated with disease progression. Subsequent investigation of a larger sample of SIV-infected monkeys confirmed this initial result. The polymorphisms were significantly associated with viral load at set-point in untreated-SIV-infected male rhesus macaques and showed a trend towards an association with survival time even after the exclusion of monkeys carrying *MHC* alleles associated with rapid or slow disease progression. One of the polymorphisms of interest is located in the 3′ UTR region of the *TLR7* mRNA, one is a silent nucleotide substitution located at amino acid position 570, and the remaining variant encodes a non-synonymous amino acid substitution (5VM) in the leader peptide. Formally, we could not distinguish which of these tightly linked polymorphisms may be responsible for the differential viral load. Furthermore, each of the three types of genetic variation seen in *TLR7* is known to affect protein expression. Mutations in the 3′UTR , for instance, can influence mRNA stability [42], silent mutations may influence translation efficiency and protein folding [43], and variations in the signal peptide can effect the transport, localization and synthesis of membrane-bound proteins [44], [45], [46].

The c.13G>A (V5M) SNP in the signal peptide represents a conservative exchange in the hydrophilic N-terminal region of the signal peptide. Using the SignalIP program [47], we found no evidence that the polymorphism would greatly influence processing of the signal peptide, although this cannot be ruled out completely without additional experimental evidence. In contrast, the 11L (*rs179008*) substitution in the human TLR7 signal peptide is potentially capable of altering signal peptide function (see supplement ref [44]). More convincing results were obtained in an analysis of RNA secondary structure. The rhesus *c.13A* allele very likely destabilizes an RNA secondary structure predicted for the *c.13G* allele. Interestingly, a strong RNA secondary structure comparable to the rhesus wild-type sequence was not predicted for the human *TLR7* signal peptide RNA sequence, and it is unlikely that the Q11L (*rs179008*) polymorphism will affect the secondary structure of the signal peptide-encoding RNA sequence. Furthermore, neither the silent mutation at amino acid position 570 nor the substitution in the 3′ UTR altered the potential secondary structure of the RNA around the respective SNPs. Based upon these predictions, the rhesus macaque *c.13A* allele is a functional candidate for the observed association with higher viral load in untreated-infected macaques [45], [46]. This result encouraged us to also investigate the influence of an adjacent polymorphism at position *−17*, which may affect the same local RNA structure and resides in a region critical for translation initiation. *In silico* analyses predicted that the effect of c.-17C>T upon RNA secondary structure was dependent upon the nucleotide present at position 13. Notably, c.-17C>T was associated with differential survival time only in *c.13G* carriers. Although translation represents a dynamic process that cannot be captured by mere sequence analyses, the coincidence of *in silico* prediction and experimental data therefore suggests that both SNPs exert a synergistic influence upon RNA structure.

It should be noted that, in whole genome screens involving human HIV-1-infected patients [48], [49], [50], the association between human *TLR7* SNPs and disease progression did not attain statistical significance after Bonferroni correction. The validity of the respective associations has therefore been questioned [51]. While variation in the *MHC* region is undoubtedly the most important host factor for determining disease progression, however, it explains only a fraction of the variability in HIV viremia [48], [49], [50], [52]. The identification of additional host gene polymorphisms contributing weakly to the variability of viremia or time to treatment initiation will require much larger cohorts and, because of the variability of the virus, the exact size of meaningful cohorts of HIV-1-infected patients may not even be known. We therefore proposed cross-species comparisons as a valuable means to validate potential host gene polymorphisms influencing the course of HIV-infection [53]. This report is the third one describing similar effects of co-localising functional genetic polymorphisms in humans and rhesus macaques even although the affected biochemical pathways may differ between the two species [53], [54]. For *TLR7*, the rhesus substitution at position 13 and −17 are more likely to influence translation efficiency whereas the human Q11L variation probably influences transport or localization. At last, however, both variants would affect TLR7 expression.

Most interestingly, the *TLR7* polymorphisms did not influence plasma viral set-point and survival time in immunized macaques. One explanation for this result could be that vaccination-induced immune reactions such as T and B cell response had a much stronger influence upon viral replication than differential *TLR7* expression.

To better define the influence of *TLR7* polymorphisms on vaccine efficacy, larger numbers of macaques treated with the same AIDS vaccine are required. Furthermore, it is paramount to investigate the exact contribution of the polymorphisms on *TLR7* expression and cytokine secretion *in vitro* and *in vivo*, in untreated and infected, and in immunized subjects. Our work highlights the dual role of TLR7 in immunodeficiency virus infection and vaccination. Assuming that the *c.13A* and *c.-17T,c.13G* variants are associated with enhanced *TLR7* expression, the data suggest that efficient triggering of TLR7 may improve AIDS vaccine efficacy while attenuating the action of TLR7 may decelerate disease progression. Finally, our results imply that it may be important to control human vaccine trials, not only for *MHC* genotype, but also for *TLR7* genotype.

## Materials and Methods

### Animals and laboratory parameters

We retrospectively analyzed genetic and phenotypic data from 237 SIV-infected rhesus macaques (*Macaca mulatta*) of Indian origin, seronegative for STLV-1 and D-type virus. These animals were housed and treated at the German Primate Research Centre (‘Deutsches Primatenzentrum’, DPZ). All relevant protocols of the DPZ comply with the German Animal Protection Act, which in turn follows European Union guidelines on the use of non-human primates for biomedical research. This includes a 12∶12 light dark schedule, provision of dry food supplemented with fresh fruit twice a day and constant water access. The monkeys were kept under permanent medical care. In cases of suffering monkeys were humanely killed. All experiments were approved by the independent governmental veterinary authority of Lower Saxony. Major characteristics of the monkeys under study and the algorithm used to define AIDS-like disease in these animals have been described before [53]. For the majority of samples viral RNA was isolated from frozen plasma samples following the MagAttract Virus Mini M48 protocol (Qiagen, Hilden, Germany). Purified SIV RNA was quantified using TaqMan-based real-time PCR on an ABI-Prism 7500 sequence detection system (Applied Biosystems) as described [55] using gag forward (5′-ACCCAGTACAACAAATAGGTGGTAACT-3′), gag reverse primer (5′-TCAATTTTACCCAGGCATTTAATGT-3′) and a fluorescent labeled probe (5′-6FAM(6-carboxyfluorescein)-TGTCCACCTGCCATTAAGCCCGAG-TAMRA(6-carboxytetramethylrhodamine-3′). Amplified viral RNA was expressed as SIV-RNA copies per mL plasma. Samples from animals infected before 2004 were analyzed using another technique [56]. Peak and set-point viremia were quantified by the RNA viral copy number at weeks 2 and 20 post infection, respectively. If in addition data at week 24 post infection were available, set-point viremia was equaled to the mean copy number at weeks 20 and 24. Note that, for some animals included in the survival analysis, no viral load data were available. Macaques were designated ‘untreated’ if they had not received any AIDS vaccine.

### *MHC*-genotyping

Monkeys were genotyped for the *MHC* as described before and classified as either ‘slow progressor’, ‘rapid progressor’ or ‘unspecified’ [34]. Survival curves used as a guide for this ‘*MHC* adjustment’ of the subsequent statistical analyses are shown in Figure S3.

### DNA sequence analysis of *TLR7*

Genomic DNA was isolated from peripheral blood or from *Herpes papio* virus-transformed B-cell lines using the Genomic DNA Isolation Kit (Qiagen, Hilden, Germany) according to the manufacturer's instructions. The rhesus *TLR7* gene was screened for polymorphisms by genomic re-sequencing of all three exons and parts of the flanking intron sequence in 36 monkeys (chrX:10564627–10566043; chrX:10582811–10589400 in the former version, rhemac2, still available at http://genome.ucsc.edu/cgi-bin/hgGateway). The primers used for nested PCR were designed using Primer3Plus [57] and are listed in Table S1. Between 25 and 50 ng of genomic DNA was used to generate the PCR template. Sequencing was performed using ABI Big Dye chemistry and the ABI3730xl Analyzer (ABI, Foster City, Calif., USA). Overlapping amplicons were sequenced to ensure that no polymorphisms in the primer binding sites were missed. Polymorphisms were searched for with the GAP software (version 4.11 of the Staden package) using the implemented SNP candidate viewer, and by visual inspection. The DNA sequences of the re-sequenced genomic regions and the polymorphisms identified in these regions are deposited in GenBank (accession number JF691587).

### Genotyping of TLR7

Three polymorphisms in the *TLR7* gene were initially genotyped by means of Sanger sequencing. For amplifying the *TLR7* c.13G>A substitution, we used primers *TLR7-13-for* (5′GGAAAATGCTGCTTCTACCATC) and *TLR7-13-rev* (5′ATGGACCAGTCTGTGAAAGGA); the silent c.1710T>A substitution was amplified with primers *TLR7-1710-for* (5′AAGTATGGGCAGACCTTGGA) and *TLR7-1710-rev* (5′CAAAAACTCCAGAAGGCAAGA). For typing 3′ UTR polymorphism c.*1604C>T, we used primers *TLR7-1604-for* (5′TCAAAGATTGAAACCTGACCAA) and *TLR7-1604-rev* (5′CAAACCACCGTACTTAACCTCA). PCR was performed using 150 ng genomic DNA and included an initial denaturation step at 94°C for 5 min, followed by 30 cycles with denaturation at 94°C for 30 s, annealing at 55°C for 30 s, and elongation at 72°C. PCR products were either precipitated with ethanol or gel-purified using standard techniques (Qiagen, Hilden, Germany). DNA sequencing was performed using dye terminator chemistry and ABI 3730/3700 technology. DNA sequences were aligned using the CLUSTALW function of the Bioedit freeware (http://www.mbio.ncsu.edu/bioedit/bioedit.html).

Alternatively genotyping was performed by means of a TaqMan-based allelic discrimination assay (ABI7500 Real Time PCR System). The reaction volume of 25 µl contained 12.5 µl ImmoMix™ (Bioline, Luckenwalde, Germany), 660 nM ROX (Invitrogen, USA) as an internal standard, 90 nM of the respective primers, 20–24 nM labelled probes and 30 ng DNA. All samples were run in triplicates. PCR was performed with an initial denaturation step at 95°C for 10 min, a pre-read step at 60°C for 1 min, followed by denaturation at 92°C for 16 s and annealing/amplification for 1 min at the indicated temperature for 40 cycles and a post read step for 1 min in at 60°C. Primers and probes for detecting the c.13G>A polymorphism were: 13-forward (5′AAAGAGAGGCAGCAAATGGGA), 13-reverse (5′AACCATCTAGCCCCAAGGAGTT), and probes FAM-5′-TTTCCAGTGTGGACACTGAAGA-3′-BBQ (20 nM) and YAK-5′-TTTCCAATGTGGACACTGAAGA-3′-BBQ (24 nM, annealing and amplification at 57°C). Oligonucleotides for detecting the c.1710T>A polymorphism were: 1710-forward (5′CAACAACCGGCTTGATTTACTC), 1710-reverse (5′GCTGGTGGAGGAAGAGATGTCA3′), and probes: FAM-5′-TTGGAAGTTCTGGATATAAGCAGT-3′-BBQ (20 nM) and YAK-5′-AATTGGAAGTACTGGATATAAGCAGT-BBQ (24 nM, annealing/amplification at 60°C). For identification of the polymorphism at position c.*1604, we used 1604-forward (5′GCAGTGCCAAAGGCTTTACTT), 1604-reverse (5′GATTAATTTGGGAATTTGTGACCCT), and the LNA-probes FAM-5′-TGAGTACACACTAAATGAATT-3′-BBQ (24 nm), YAK-5′-TGTGAGTATACACTAAATGAAT-3′-BBQ (24 nmol, annealing/amplification at 59°C). The c.-17C>T polymorphism was typed using −17-forward (5′ATCTCAAGCTGATCTTGGCACC), −17 reverse (5′GGTTTCAGTTGTTTCCACAAAGATT), and LNA-probes FAM-5′TCCAAAATGGAATGTAGAGGTCTG-3′-BBQ, YAK-5′-TCCAGAATGGAATGTAGAGGTCTG-3′-BBQ (20 nM each, annealing/amplification at 60°C). Primers and probes were designed and synthesized by TIPMolbiol, Berlin, Germany. Allele calling was performed on the basis of the amplification and post-amplification plate reads.

### Statistical analysis

Survival analysis was performed using the LIFETEST and LIFEREG procedures of the SAS software package (SAS version 9.2, SAS Inc., Cary, NC). Differences between genotype-specific survival curves were assessed for statistical significance using a log-rank test with the appropriate degrees of freedom. Unbalanced analysis of variance (ANOVA) of log-transformed viral load data was performed using SAS procedure GLM. Kaplan-Meier survival curves were prepared with SigmaPlot 8.0 (IBM SPPS Inc. Somers, NY). All other statistical analyses were carried out using the FREQ procedure of SAS.

### Analysis of RNA secondary structure

Two online prediction tools were employed for RNA secondary structure analysis, namely the Contrafold option in CentroidFold [58] and RNAfold from the Vienna RNA Websuite [59]. Secondary structure prediction of the region encoding the signal peptide covered nucleotide positions 1 to 78 while positions −136 to 78 were involved in the analysis of the c.-17C>T polymorphism. For the remaining two polymorphisms, 30 base pairs both upstream and downstream of the site of interest were included.

## Supporting Information

Figure S1 **Alignment of rhesus and human** ***TLR7*** **gene DNA sequences.** The rhesus genomic DNA and refseq mRNA (acc. no. NM_001130426) sequences were taken from the USCS genome browser (http://genome.ucsc.edu/). Untranslated regions are still hypothetical. Coding sequences are shown in black, untranslated regions are given in purple. Polymorphic positions in the rhesus sequence are marked.
(DOC)

Figure S2 **Alignment of rhesus and human TLR7 amino acid sequence.** Amino acid exchanges between human and rhesus are shown in bold, identity between deduced amino acid residues is depicted by a dot. Signal peptide, transmembrane and TIR domain are marked by red letters. Polymorphic sites in the rhesus TLR7 sequence are annotated (green for synonymous, blue for non-synonymous nucleotide exchanges).
(DOC)

Figure S3 **Kaplan Meier Survival curves of SIV-infected male rhesus macaques of Indian origin stratified by** ***MHC*** **genotype.** ‘*MHC* adjusted’ refers to the exclusion of animals carrying either Mamu-A1*001, B*008 or an *MHC* genotype known to be associated with rapid disease progression. A: untreated SIV-infected macaques (p<0.01). B: immunized SIV-infected macaques (p<0.01).
(TIF)

Table S1 Primers used for PCR amplification of the rhesus macaque TLR7 gene region.
(DOC)
