# Supplementary material for: Association of TLR7 Variants with AIDS-Like Disease and AIDS Vaccine Efficacy in Rhesus Macaques
Source: PLoS One. 2011 Oct 13;6(10):e25474. doi: 10.1371/journal.pone.0025474 (PMC3192768; doi:10.1371/journal.pone.0025474)
Supplement: Figure S1 — Alignment of rhesus and human TLR7 gene DNA sequences. The rhesus genomic DNA and refseq mRNA (acc. no. NM_001130426) sequences were taken from the USCS genome browser (http://genome.ucsc.edu/). Untranslated regions are still hypothetical. Coding sequences are shown in black, untranslated regions are given in purple. Polymorphic positions in the rhesus sequence are marked. (DOC) [file pone.0025474.s001.doc]

**Figure S1: Alignment of rhesus and human *TLR7* gene DNA sequences**

**Exon 1**

Rhesus GAAGACTCGAGAT-TAGGATCACTCCATACCATCAAGAAAG

human ........**C....A..............G............**

**Exon 2**

rhesus TTGATGCTATTGGGTCCATCTCAAGCTGATCTTGGCACCTCTCATGCTCTGCTCTCTTCA

human ..............**C**..............................................

-**17 (C→T)** +1

rhesus ACCAGACCTCTACATTCCATT**C**TGGAAGAAGACTAAAAATG

human .....................T...................

**Exon 3**

**V5M (G→A)**

10 20 30 40 50 60 70 80

rhesus ATGTTTCCA**G**TGTGGACACTGAAGAGACAAATTCTTATCCTTTTTAACATAATCCTAATTTCCAAACTCCTTGGGGCTAG

human G........A......................................................................

90 100 110 120 130 140 150 160

rhesus ATGGTTTCCTAAAACTCTGCCCTGTGATGTCACTCTGGATGTTTCAAAGAACCATGTGATCGTGGACTGCACAGACAAGC

human ...........................................C....................................

**T68A(A→G)**

170 180 190 200 210 220 230 240

rhesus ATTTGACAGAAATTCCTGGAGGTATTCCCACCAACACT**A**CGAACCTCACCCTCACCATTAACCACATACCAGACATCTCC

human ...............................G.....C..........................................

250 260 270 280 290 300 310 320

rhesus CCAGCGTCCTTTCACAGACTGGTCCATCTGGTAGAGATCGATTTCAGATGCAACTGTGTACCTATTCGATTGGGGTCAAA

human ......................A............................................C.C..........

330 340 350 360 370 380 390 400

rhesus AAGCAACATGTGCCCCAGGAGGCTGCAGATTAAACCCAGAAGCTTTAGTGGACTCACTTATTTAAAATCCCTTTACCTGG

human ..A..........AT..A..............................................................

410 420 430 440 450 460 470 480

rhesus ATGGAAACCAGCTTCTAGAGATACCGCAGGGCCTTCCACCCAGCTTACAGCTTCTCAGCCTTGAGGCCAACAACATCTTT

human .............A....................C..G..T.......................................

490 500 510 520 530 540 550 560

rhesus TCCATCAGAAAAGAGAATCTAACAGAACTGGCCAACATAGAAATACTCTACCTGGGCCAAAACTGTTATTATCGAAATCC

human ................................................................................

570 580 590 600 610 620 630 640

rhesus TTGTTATGTTTCATATTCAATAGAAAAAGATGCCTTCCTAAACTTGACAAAGTTAAAAGTGCTCTCCCTGAAAGATAACA

human ........................G.......................................................

650 660 670 680 690 700 710 720

rhesus ATGTCACAACCGTCCCTACTGTTTTGCCATCTACTTTAACAGAACTATATCTCTACAACAACATGATTGCAGAAATCCAA

human ........G..............................................................G........

730 740 750 760 770 780 790 800

rhesus GAAGATGATTTTAATAACCTCAACCAATTACAAATTCTTGACCTAAGTGGAAATTGCCCTCGTTGTTATAATGCCCCATT

human ................................................................................

810 820 830 840 850 860 870 880

rhesus TCCTTGTACGCCATGTAAAAATAATTCTCCCCTACAGATCCCTGTAAATGCTTTTGATGCGCTGACAGAATTAAAAGTTT

human .......G....G...................................................................

890 900 910 920 930 940 950 960

rhesus TACGTCTACACAGTAACTCTCTTCAGCATGTGCCCCCAAGATGGTTTAAGAACATCAACAATCTCCAGGAACTAGATCTG

human .............................................................A...........G......

970 980 990 1000 1010 1020 1030 1040

rhesus TCCCAAAACTTCTTGGCCAAAGAAATTGGGGATGCCAAATTTCTGCATTTTCTCCCCAACCTCATCCAATTGGATCTGTC

human ...................................C......................G.....................

1050 1060 1070 1080 1090 1100 1110 1120

rhesus TTTCAATTTTGAACTTCAGGTCTATCGTGCATCTATGAATCTATCACAAGCATTTTCTTCACTGAAAAGCCTGAAAATTC

human ................................................................................

1130 1140 1150 1160 1170 1180 1190 1200

rhesus TGCGGATCAGAGGATATGTCTTCAAAGAGCTGAAAAGCTTTAACCTCTCTCCATTACATAATCTTCAAAATCTTGAAGTT

human ......................T......T...................G..............................

1210 1220 1230 1240 1250 1260 1270 1280

rhesus CTTGATCTTGGTACTAACTTTATAAAAATTGCTAACCTCAGCATGTTTAAACAATTTAAAAGATTGAAAGTCATAGATCT

human ...........C...................................................C................

1290 1300 1310 1320 1330 1340 1350 1360

rhesus TTCAGTGAATAAAATATCACCTTCAGGAGATTCAAGTGAAGTTGGCTTCTGCTCAAATGCCAGAACTTCTGTAGAAAGTT

human ................................................................................

1370 1380 1390 1400 1410 1420 1430 1440

rhesus ATGAACCCCAGGTCCTGGAACAATTATATTATTTCAGATATGATAAGTATGCAAGGAGTTGCAGGTTCAAAAACAAAGAG

human ..........................C.....................................A...............

1450 1460 1470 1480 1490 1500 1510 1520

rhesus GCTTCTTTCACGTCTGTTAATGAAAGCTGCTACAAGTATGGGCAGACCTTGGATCTAAGTAAAAATAGTATATTTTTTAT

human ..........T...................................................................G.

1530 1540 1550 1560 1570 1580 1590 1600

rhesus CAAGTCCTCTGATTTTCAGCATCTTTCTTTCCTCAAATGCCTGAATTTGTCAGGAAATCTCATTAGCCAAACTCTTAATG

human ..............................................C.................................

1610 1620 1630 1640 1650 1660 1670 1680

rhesus GCAGTGAATTCCAACCTTTAGCAGAGCTGAGATATTTGGACTTCTCCAACAACCGGCTTGATTTACTCCATTCAACAGCA

human ................................................................................

**V570V (T→A)**

1690 1700 1710 1720 1730 1740 1750 1760

rhesus TTTGAAGAGCTTCGCAAATTGGAAGT**T**CTGGATATAAGCAGTAATAGCCATTATTTTCAATCAGAAGGAATTACTCATAT

human .............A....C.............................................................

1770 1780 1790 1800 1810 1820 1830 1840

rhesus GCTAAACTTTACCAAGAACCTAAAGGTTCTGCAGAAACTGATGATGAACGACAATGACATCTCTTCCTCCACCAGCAGGA

human ................................................................................

1850 1860 1870 1880 1890 1900 1910 1920

rhesus CCATGGAGAGTGAGTCTCTTAGAACTCTGGAATTCAGAGGAAATCACTTAGATGTTTTATGGAGAGATGGTGATAACAGA

human ...................................................................A............

1930 1940 1950 1960 1970 1980 1990 2000

rhesus TACTTACAATTATTCAAGAATCTGCTAAAATTAGAGGAATTAGACATCTCTAAAAATTCCCTAAGTTTCTTGCCTTCTGG

human ................................................................................

2010 2020 2030 2040 2050 2060 2070 2080

rhesus AGTTTTTGATGGTATGCCTCCAAATCTAAAGAATCTCTCTTTGGCCAAAAATGGGCTCAAATCTTTCATTTGGGAAAAAC

human ....................................................................G....A.G....

2090 2100 2110 2120 2130 2140 2150 2160

rhesus TCCGTTATCTAAAGAACCTGGAAACTTTGGACCTCAGCCACAACCAACTGACGACTGTCCCTGAGAGATTATCCAACTGT

human ...AG.G.............................................C...........................

2170 2180 2190 2200 2210 2220 2230 2240

rhesus TCCAGAAGCCTCAAGAATCTGATTCTTAAGAATAATCAAATCAGGAGTCTGACGAAGTATTTTCTACAAGATGCCTTCCA

human ................................................................................

2250 2260 2270 2280 2290 2300 2310 2320

rhesus GTTGCGATATCTGGATCTCAGCTCAAATAAAATCCAGATGATCCAAAAGACCAGCTTCCCAGAAAATGTCCTCAACAATC

human ................................................................................

**R784R (G→A)**

2330 2340 2350 2360 2370 2380 2390 2400

rhesus TGAAGATGTTGCTTTTGCATCATAATCG**G**TTTTTGTGCACCTGTGATGCTGTGTGGTTTGTCTGGTGGGTTAACCATACG

human ............................G...C...............................................

2410 2420 2430 2440 2450 2460 2470 2480

rhesus GAGGTGACTATTCCTTACCTGGCCACAGACGTGACTTGCGTGGGGCCAGGAGCACACAAGGGCCAGAGTGTGATCTCCCT

human .............................T........T..........................A..............

2490 2500 2510 2520 2530 2540 2550 2560

rhesus GGATCTGTATACCTGTGAGTTAGATCTGACTAACCTGATTCTGTTCTCACTTTCCATATCTGTATCTCTCTTTCTCATGG

human .........C......................................................................

2570 2580 2590 2600 2610 2620 2630 2640

rhesus TGATGATGACAGCAAGTCACCTCTATTTCTGGGATGTGTGGTATATTTACCATTTCTGTAAGGCCAAGATAAAGGGGTAT

human ................................................................................

2650 2660 2670 2680 2690 2700 2710 2720

rhesus CAGCGTCTAATATCACCAGACTGTTGCTATGATGCTTTCATTGTGTATGACACTAAAGACCCAGCTGTGACAGAGTGGGT

human ......................................T................................C........

2730 2740 2750 2760 2770 2780 2790 2800

rhesus TTTGGCTGAGCTGGTGGCCAAACTGGAAGACCCAAGAGAGAAACATTTTAATTTATGTCTCGAGGAAAGGGACTGGTTAC

human ................................................................................

2810 2820 2830 2840 2850 2860 2870 2880

rhesus CAGGGCAGCCAGTTCTGGAAAACCTTTCCCAGAGCATACAGCTTAGCAAAAAGACAGTGTTTGTGATGACAGACAAGTAT

human ................................................................................

2890 2900 2910 2920 2930 2940 2950 2960

rhesus GCAAAGACCGAAAATTTTAAGATAGCATTTTACTTGTCCCATCAGAGGCTCATGGATGAAAAAGTTGATGTGATTATCTT

human ........T.......................................................................

2970 2980 2990 3000 3010 3020 3030 3040

rhesus GATATTTCTTGAGAAGCCCTTTCAGAAGTCCAAGTTCCTCCAGCTCCGGAAAAGACTCTGTGGGAGTTCTGTCCTTGAGT

human ......................................................G.........................

3050 3060 3070 3080 3090 3100 3110 3120

rhesus GGCCAACAAACCCGCAGGCTCACCCATACTTCTGGCAGTGTCTAAAGAACGCCCTGGCCACAGACAATCACGTGGCCTAT

human ................A.....................................................T.........

3130 3140 3150 3160 3170 3180 3190 3200

rhesus AGTCAGGTGTTCAAGGAAACGGTCTAGCCCTTCTTTGCAAAACACGACTGCCTAGCTTACCAAGGAGATGCCTGGTGGTT

human .............................................A.........T............G......CT...

3210 3220 3230 3240 3250 3260 3270 3280

rhesus TAAATTGTTTTCATGTATGTCACACCAAAAGCGTGTTTTGAAATTCTTCAAGAAATGAGATTGCCCATATTTCGGGGGAG

human ..............A...A......................................................A......

3290 3300 3310 3320 3330 3340 3350 3360

rhesus TCACCAACATCTGTCACAGGAGTTGGAAAGATGGAATTTATATAATGCATTGAGTCTTCTTTCCTATGTCTCTGTGTCTC

human T.......G.........................GG..............CA...........T...C............

3370 3380 3390 3400 3410 3420 3430 3440

rhesus TATTTGCACTTGAGTCTCTCCCCTCAGCTCTTGTATAAAATACTGTTGGGAAAGGAGTGCCAAGTAAAAAACACAGGGCT

human ....................A.........C....----------------..A.....G.............TG.....

3450 3460 3470 3480 3490 3500 3510 3520

rhesus CTGATGCTCCTGTAATTGTGATGATTAAATATACACACAATCATGACATTGAGGAGAACTGCATTTCTACCCTTAAAAAG

human .....T................A..............................A..........................

***437 (G→A)**

3530 3540 3550 3560 3570 3580 3590 3600

rhesus TACTGGTATATACAGAAATAGGGTTTAAAAAAAGCTCAAGCTCTGTCTATATGAGACCAAAAT**G**TACTAGAGTTAGTTTA

human .........................A.......-..........C...................................

3610 3620 3630 3640 3650 3660 3670 3680

rhesus GTGAAATAAAAACCCAGTCAACTGGCTGGGCACGGTGGCTCATGCTTGTAATCCCAGCACTTTGGGAGGCCGAGGCGGGT

human ............A.......G.....C.....T...........................................A...

3690 3700 3710 3720 3730 3740 3750 3760

rhesus GGATCACGAGATCAGGAGTTCAAGACCAGTCTGGCCAACACGGTGAAACCCTGTCTCTGCTAAAAATACAAAAATTAGCT

human ..........G.........TG..................T..........C....G.A.....................

3770 3780 3790 3800 3810 3820 3830 3840

rhesus GCGCGTGGTGGCAGGTGCCTATAATCCCAGCTACTTGGGAGGCTGAGGCAGGAGAATTGCTTGAACCTGGGAGGCGGAGG

human .C.........TG.......G....................................C.........C......T.....

***236 (A→G)**

3850 3860 3870 3880 3890 3900 3910

rhesus TGGCAGTGAGCCAAGATTGTGCCACTGCACTCCAGCCTGGGC**A**ACAGAGCTAGACTCTGTCTCAA-----AAAACAAACA

human ............A....CAC.........A.G.....C..................G.C..AA..GAACA....A...A.

3920 3930 3940 3950 3960 3970 3980 3990

rhesus AACA-AACAAAACCCAGTCAACTTCTTAACCAATTGCTTCTGTGACATCCAGGGCCCCGTTCTGTGCAGATCGAGTGTGG

human ....C..A.....T......G...................C...T.............A............T........

## *867(T→C) *908_1911delGGCT

4000 4010 4020 4030 4040 4050 4060 4070

rhesus GCACCACACAGGTGGTTGC**T**GCTTCAGTGCTTCCTGCTCTTTTCCCTTGGGCCTGCTCCT**GGCT**TCCATAGGGAAACAGC

human ...........................................T.............T....G..................

## *941 (A→G)

## 4080 4090 4100 4110 4120 4130 4140 4150

rhesus GAGAAAGAAAGAC**A**CATCCTTACCATAAACGGATATGGTCCACCTACAAATGGAAAAATATTTAAATGATCTGCCTTTAT

human A............................T.C...................A............................

4160 4170 4180 4190 4200 4210 4220 4230

rhesus ACAAAGTGATATTCTCTACCTTTGATAATTTACCTGCTTAAATGTTTTTATCTGTACTGCAAAGTACTGTATCCAAAGTA

human ......................................................T.........................

4240 4250 4260 4270 4280 4290 4300 4310

rhesus AAATTTCCTCATCCAATATCTTTCAAACTGTTTTGTTAACTAATGCCATATATTTGTAAATACCTGCACGCTTGATACAG

human ...........................................................G..T......A..........

4320 4330 4340 4350 4360 4370 4380 4390

rhesus CAATGTTAGATGGTTTTGACGGTAAGCCCTAAAGGAGGACTCCGAGAGTGTGTATTTATCTATACTTTTATCAGAGATGA

human ...T...............T.....A.................G...............T....G...............

4400 4410 4420 4430 4440 4450 4460 4470

rhesus CAATTATTTGAATGCCAATTATATGGATTCCTTTCATTTTTTGCTGGGGGATGGGAGAAGAAACCAAAGTTTATAGACCT

human ...............................................A................................

4480 4490 4500 4510 4520 4530 4540 4550

rhesus TCACATTGAGAAAGCTTCAGTTTTGAACTTCAACTATCAGATTCAAAAACAACAGAAAGAACCAAGACATTCTTAAGACA

human ................................G.............................................TG

4560 4570 4580 4590 4600 4610 4620 4630

rhesus CATGTACTTTCAACTGGGTATAAATTCATGAGTTCAAAGATTGAAACCTGACCAATTTGCTTTATTTCATGGAAGAAGTG

human .C..........G...................................................................

4640 4650 4660 4670 4680 4690 4700 4710

rhesus ATCTACAATGGTGTTTGTGCCATTTGGAAAACAGCGTGCATGTGTTCAAGCCTGTGATTGAAGA---CATATTTTCCTCA

human ........A............................................TA.....GG..TGT.G...........

***1604(C→T)**

4720 4730 4740 4750 4760 4770 4780 4790

rhesus TATGTGGCAGTGCCAAAGGCTTTACTTTACCTGTGAGTA**C**ACACTAAATGAATTATTTCCAAAGGACATTTAATCAGTAA

human CG.......A....................................T...............C.T...........A...

***1681 (A→G)**

4800 4810 4820 4830 4840 4850 4860

rhesus GGGTCACAAATTCCCAAATTAATCTCTGGAATAAAT**A**GAGAGGTAATTGAATTGCTGGAGTCAACT

human ...................C............................A...........C.....
