# Supplementary material for: Association of TLR7 Variants with AIDS-Like Disease and AIDS Vaccine Efficacy in Rhesus Macaques
Source: PLoS One. 2011 Oct 13;6(10):e25474. doi: 10.1371/journal.pone.0025474 (PMC3192768; doi:10.1371/journal.pone.0025474)
Supplement: Figure S2 — Alignment of rhesus and human TLR7 amino acid sequence. Amino acid exchanges between human and rhesus are shown in bold, identity between deduced amino acid residues is depicted by a dot. Signal peptide, transmembrane and TIR domain are marked by red letters. Polymorphic sites in the rhesus TLR7 sequence are annotated (green for synonymous, blue for non-synonymous nucleotide exchanges). (DOC) [file pone.0025474.s002.doc]

**Figure S2: Alignment of rhesus and human TLR7 amino acid sequences**

**Signal Peptide**

**V5M**

rhesus M**M**FP**V**WTLKRQILILFNIILISKLLGARWFPKTLPCDV**T**LDVSKNHVIVDCTDKHLTEIP 60

human .**V**..**M.................................P.....................**

**T68A**

rhesus GGIPTNT**T**NLTLTINHIPDISPASFHRL**V**HLVEIDFRCNCVPI**R**LGSK**S**NMC**PR**RLQIKP 120

human ............................**D..............P....N...IK**......

rhesus RSFSGLTYLKSLYLDGNQLLEIPQGLPPSLQLLSLEANNIFSIRKENLTELANIEILYLG 180

human ............................................................

rhesus QNCYYRNPCYVSYSIEKDAFLNLTKLKVLSLKDNNVT**T**VPTVLPSTLTELYLYNNMIA**E**I 240

human .....................................**A....................K.**

rhesus QEDDFNNLNQLQILDLSGNCPRCYNAPFPC**T**PCKNNSPLQIPVNAFDALTELKVLRLHSN 300

human ..............................**A.............................**

rhesus SLQHVPPRWFKNIN**N**LQELDLSQNFLAKEIGDAKFLHFLP**N**LIQLDLSFNFELQVYRASM 360

human ..............**K**.........................**S**...................

rhesus NLSQAFSSLKSLKILRIRGYVFKELKSFNLSPLHNLQNLEVLDLGTNFIKIANLSMFKQF 420

human ............................................................

rhesus KRLKVIDLSVNKISPSGDSSEVGFCSNARTSVESYEPQVLEQL**Y**YFRYDKYARSCRFKNK 480

human ...........................................**H................**

rhesus EASF**T**SVNESCYKYGQTLDLSKNSIFF**I**KSSDFQHLSFLKCLNLSGNLISQTLNGSEFQP 540

human ....**M**......................**V**................................

**V570V**

rhesus LAELRYLDFSNNRLDLLHSTAFEEL**R**KLE**V**LDISSNSHYFQSEGITHMLNFTKNLKVLQK 600

human .........................**H..................................**

rhesus LMMNDNDISSSTSRTMESESLRTLEFRGNHLDVLWR**D**GDNRYLQLFKNLLKLEELDISKN 660

human ...................................**.E.......................**

rhesus SLSFLPSGVFDGMPPNLKNLSLAKNGLKSF**I**W**E**KL**RY**LKNLETLDLSHNQLTTVPERLSN 720

human ..............................**S.K..QC.......................**

rhesus CSRSLKNLILKNNQIRSLTKYFLQDAFQLRYLDLSSNKIQMIQKTSFPENVLNNLKMLLL 780

human ............................................................

**R784R**

rhesus HHN**R**FLCTCDAVWFVWWVNHTEVTIPYLATDVTCVGPGAHKGQSVISLDLYTCELDLTNL 840

human ............................................................

**Transmembrane Region**

rhesus ILFSLSISVSLFLMVMMTASHLYFWDVWYIYHFCKAKIKGYQRLISPDCCYDAFIVYDTK 900

human ............................................................

**Toll/IL-1 receptor (TIR) domain**

rhesus DPAVTEWVLAELVAKLEDPREKHFNLCLEERDWLPGQPVLENLSQSIQLSKKTVFVMTDK 960

human ............................................................

rhesus YAKTENFKIAFYLSHQRLMDEKVDVIILIFLEKPFQKSKFLQLRKRLCGSSVLEWPTNPQ 1020

human ............................................................

rhesus AHPYFWQCLKNALATDNHVAYSQVFKETV 1049

human .............................
