# Supplementary figures and images for: Association of TLR7 Variants with AIDS-Like Disease and AIDS Vaccine Efficacy in Rhesus Macaques
Source: PLoS One. 2011 Oct 13;6(10):e25474. doi: 10.1371/journal.pone.0025474 (PMC3192768; doi:10.1371/journal.pone.0025474)

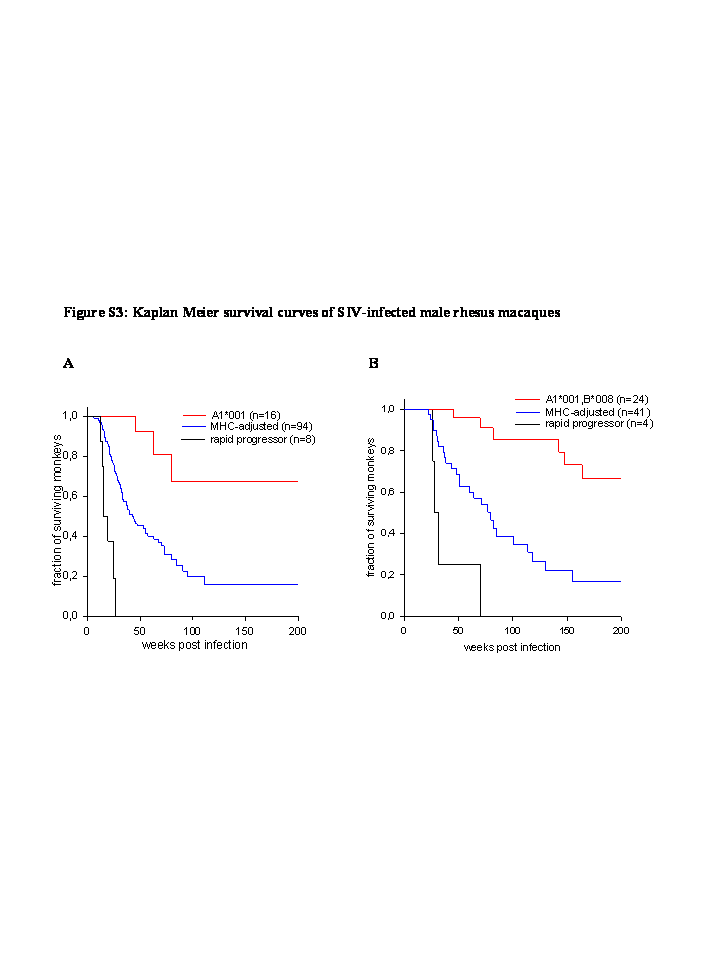

Supplement: Figure S3 — Kaplan Meier Survival curves of SIV-infected male rhesus macaques of Indian origin stratified by MHC genotype. ‘MHC adjusted’ refers to the exclusion of animals carrying either Mamu-A1*001, B*008 or an MHC genotype known to be associated with rapid disease progression. A: untreated SIV-infected macaques (p<0.01). B: immunized SIV-infected macaques (p<0.01). (TIF) [file pone.0025474.s003.tif]
