# Supplementary material for: Association of TLR7 Variants with AIDS-Like Disease and AIDS Vaccine Efficacy in Rhesus Macaques
Source: PLoS One. 2011 Oct 13;6(10):e25474. doi: 10.1371/journal.pone.0025474 (PMC3192768; doi:10.1371/journal.pone.0025474)
Supplement: Table S1 — Primers used for PCR amplification of the rhesus macaque TLR7 gene region. (DOC) [file pone.0025474.s004.doc]

**Table S1: Primers used for PCR amplification of the rhesus macaque TLR7 gene region**

| **exon 1 and exon 2 primers:** |  |
| --- | --- |
| tlr7_F1_1 | TCCTCTCCAGCTGGGTCTAA |
| tlr7_F1_2 | ATGGGTGGCAGGAAGTGTAG |
| tlr7_F1_3 | AATAAGAGGAACCCGGTGCT |
| tlr7_F1_4 | ATGCTGCGATCATTGCTTTA |
| tlr7_F1_5 | TCCAGTCTACCTAACTTCGAGGA |
| tlr7_F1_6 | TTGCTATGAGCCTGCCTACA |
| tlr7_F1_7 | CCCCCTTCATTAATGCCAAT |
| tlr7_F1_8 | TGGCAAGAGCAATGTTGAAG |
|  |  |
| **exon 3 primers**: |  |
| tlr7_F2_1 | AGTGCCAGAATGGCATGAAT |
| tlr7_F2_2 | CAGGCCTTGTTTCCAACTGT |
| tlr7_F2_3 | TGCTGCTTCTACCATCTTGAAA |
| tlr7_F2_4 | AGGTTCGTAGTGTTGGTGGG |
| tlr7_F2_5 | ACTCCTTGGGGCTAGATGGT |
| tlr7_F2_6 | TTCTGTCAGCGCATCAAAAG |
| tlr7_F2_7 | CCTCAAGGCTGAGAAGCTGT |
| tlr7_F2_8 | TTACCTGGATGGAAACCAGC |
| tlr7_F3_1 | CGTCCCTACTGTTTTGCCAT |
| tlr7_F3_2 | TCCTCTGATCCGCAGAATTT |
| tlr7_F3_3 | TTGAACTTCAGGTCTATCGTGC |
| tlr7_F3_4 | AGATCCAAGGTCTGCCCATA |
| tlr7_F3_5 | ATGCAAGGAGTTGCAGGTTC |
| tlr7_F3_6 | CGGAGTTTTTCCCAAATGAA |
| tlr7_F3_7 | CTGCTGGTGGAGGAAGAGAT |
| tlr7_F3_8 | CAACCGGCTTGATTTACTCC |
| tlr7_F4_1 | TGCCTTCTGGAGTTTTTGATG |
| tlr7_F4_2 | GTCTGTGGCCAGGTAAGGAA |
| tlr7_F4_3 | CAGCTTCCCAGAAAATGTCC |
| tlr7_F4_4 | GCCAAAACCCACTCTGTCAC |
| tlr7_F4_5 | GGCCAAGATAAAGGGGTATCA |
| tlr7_F4_6 | GAGCTGAGGGGAGAGACTCA |
| tlr7_F4_7 | CACAGAGTCTTTTCCGGAGC |
| tlr7_F4_8 | TTGTCCCATCAGAGGCTCAT |
| tlr7_F5_1 | CACCAAAAGCGTGTTTTGAA |
| tlr7_F5_2 | TTGGTCTCATATAGACAGAGCTTGA |
| tlr7_F5_3 | TGGGAAAGGAGTGCCAAGTA |
| tlr7_F5_4 | CCTGGATGTCACAGAAGCAA |
| tlr7_F5_5 | CCTGGGCAACAGAGCTAGAC |
| tlr7_F5_6 | TTTTCCAAATGGCACAAACA |
| tlr7_F5_7 | CATTGCTGTATCAAGCGTGC |
| tlr7_F5_8 | TGACGGTAAGCCCTAAAGGA |
| tlr7_F6_1 | GGATGGGAGAAGAAACCAAA |
| tlr7_F6_2 | GCCATTATCCTTGTTCTCACG |
| tlr7_F6_3 | AAGGGTCACAAATTCCCAAA |
| tlr7_F6_4 | ACATTCAGAGCTTTGTGGGG |
| tlr7_F6_5 | TGAGGTTAAGTACGGTGGTTTG |
| tlr7_F6_6 | TCCTCTTGTTCACCCGAGTT |
| tlr7_F6_7 | TCGGGGCTACAATCAGACTC |
| tlr7_F6_8 | GCAGGGCAAATGTTATGGAT |
